# Neuronal Correlates of Risk-Seeking Attitudes to Anticipated Losses in Binge Drinkers

**DOI:** 10.1016/j.biopsych.2013.11.028

**Published:** 2014-11-01

**Authors:** Yulia Worbe, Michael Irvine, Iris Lange, Prantik Kundu, Nicholas A. Howell, Neil A. Harrison, Edward T. Bullmore, Trevor W. Robbins, Valerie Voon

**Affiliations:** a Behavioural and Clinical Neurosciences Institute, University of Cambridge, Cambridge, United Kingdom; b Department of Psychiatry, Addenbrooke’s Hospital, University of Cambridge, Cambridge, United Kingdom; c Brighton and Sussex Medical School, University of Sussex, Brighton; d Cambridgeshire & Peterborough National Health Service Foundation Trust, Cambridge, United Kingdom; e GlaxoSmithKline, Clinical Unit Cambridge, Cambridge, United Kingdom

**Keywords:** Binge drinking, feedback sensitivity, functional MRI, loss anticipation, risk-seeking behavior

## Abstract

**Background:**

Abnormal decision making under risk is associated with a number of psychiatric disorders. Here, we focus on binge drinkers (BD), characterized by repeated episodes of heavy alcohol intoxication. Previous studies suggest a decreased sensitivity to aversive conditioning in BD. Here, we asked whether BD might be characterized by enhanced risk seeking related to decreased sensitivity to the anticipation of negative outcomes.

**Methods:**

Using an anticipatory risk-taking task (40 BD and 70 healthy volunteers) and an adapted version of this task for functional magnetic resonance imaging (21 BD and 21 healthy volunteers), we assessed sensitivity to reward and loss across risk probabilities.

**Results:**

In the behavioral task, BD showed a higher number of risky choices in high-risk losses. In the neuroimaging task, the high-risk attitude in the loss condition was associated with greater activity in dorsolateral prefrontal, lateral orbitofrontal, and superior parietal cortices in BD. Explicit exposure of BD to the probability and magnitude of loss, via introduction of feedback, resulted in a subsequent decrease in risky choices. This change in risk attitude in BD was associated with greater activity in inferior frontal gyrus, which also correlated with the percentage of decrease in risky choices after feedback presentation, suggesting a possible role for cognitive control toward risk-seeking attitudes.

**Conclusions:**

Our findings suggest that a decrease in sensitivity to the anticipation of high-risk negative outcomes might underlie BD behavior. Presentation of explicit feedback of probability and loss in BD can potentially modify risk-taking attitudes, which have important public health implications and suggest possible therapeutic targets.

The evaluation of risk is ubiquitous in daily life. Whether we choose to change careers or go snowboarding with friends, we weigh our options based on potential probabilities of good or harm. Prospect theory (1) and evidence from studies in healthy individuals suggest that decisions taken under risky conditions are commonly based on our anticipation of the probabilistic outcomes and influenced by our personal experiences (2) and biological traits 3, 4.

Most healthy individuals are risk averse. A neuronal network including dorsolateral prefrontal, orbitofrontal, insular, parietal, and posterior cingulate cortices and dopaminergic midbrain neurons 5, 6, 7, 8, 9 are suggested to mediate the judgment of choice riskiness.

Risk seeking is associated with a number of disorders such as substance and behavioral addictions, including pathological gambling in the general population, and related to dopaminergic medications in Parkinson’s disease 10, 11, 12. Reduced reward discrimination and impaired neuronal risk signaling 13, 14, 15 are related to maladaptive risk seeking. Sensitivity to learning from negative outcomes has also been shown to underlie pathologic decisions under risky conditions. For instance, stimulant-dependent subjects have been shown to be impaired at learning to avoid negative feedback in a modified Iowa Gambling Task (16). Harmful alcohol users without a history of dependence also demonstrated impaired inhibition of prepotent responses to reward in the presence of immediate monetary punishment for failure in a monetary incentive go/no go task (17).

In this study, we use a risk-taking task in which subjects choose between a sure choice and a gamble, focusing on the anticipation of risky outcomes across a range of probabilities. We focus on binge drinkers (BD), characterized by repeated episodes of heavy alcohol intoxication (18), a recognized risk factor for the development of alcohol dependency (19). Binge drinking is a major public health issue associated with marked negative physical, emotional, and financial consequences 18, 20, 21 and limited preventive interventions (22).

Acute alcohol administration has been shown to reduce response avoidance for aversive consequences (23). In rodent models of BD, repeated ethanol exposure and withdrawal induced impairment in aversive reinforcement, along with reduced long-term potentiation in limbic brain structures 24, 25, 26. Human BD subjects also showed reduced sensitivity to aversive reinforcement (26).

Consequently, in this study, we asked whether BD might be characterized by enhanced risk seeking related to decreased sensitivity to the anticipation of negative outcomes. To this end, we first tested BD using an anticipatory risk-taking task without feedback to assess sensitivity to reward and loss across probabilities. We demonstrated that BD had a greater risk-seeking attitude specifically to high-risk losses. We then developed a task for functional magnetic resonance imaging focusing on the anticipation of risky losses without feedback. We hypothesized that BD would be more risk seeking in the loss domain and that this would be associated with abnormal activation of regions associated with the representation of risk. Finally, we introduced an intervention of experiential exposure to the explicit probability and monetary loss feedback of this high-risk loss task to test the capacity to modulate risk-taking choices.

## Methods and Materials

### Participant’s Inclusion Criteria

The study was approved by the Cambridge University Research Ethics Committee. Participants were recruited from community and university-based advertisements and gave informed consent before the study. The inclusion criteria were age above 18 years, no history of neurological or psychiatric disorders as assessed with the Mini-International Neuropsychiatric Interview (27), and no regular use of drugs (except nicotine). The BD were included if they fulfilled the National Institute of Alcohol Abuse and Alcoholism diagnostic criteria (28) of >8 alcohol units consumed for male subjects (>6 for female subjects) in a 2-hour period, at least once a week, over a period of 3 months before testing. Subjects also reported the intention to get drunk. Participants first completed a behavioral version of the task and a subset returned for the functional magnetic resonance imaging arm of the study. Volunteers participating in the neuroimaging arm were also screened for magnetic resonance imaging contraindications. Before testing, all participants were screened using an alcohol breathalyzer test and urine drug screen and excluded if the tests were positive.

### Behavioral Task

**Figure 1:**
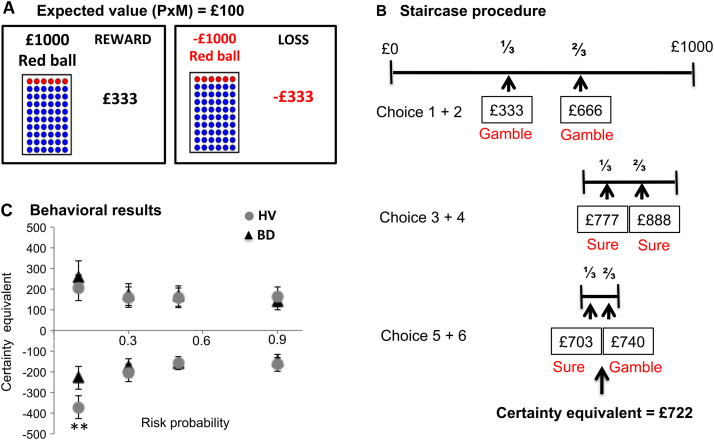
**(A)** Behavioral risk-choice task: the proportion of red balls in the jar represents the chance to win (in reward–on the left) or to lose (in loss–on the right) on each session of the task. **(B)** A staircase procedure in which the amount of the sure option was adjusted depending on choices in the previous trial. If the subject chose the gamble, the amount of two further sure options was calculated as follows: Sure amount = lower magnitude of sure amount in previous trial + (higher − lower magnitude of sure amount in previous trial) × 1/3 (× 2/3 in the consecutive trial), producing the increase of sure amount in the next trials. In the case of sure choice, the amount of consecutive sure options was calculated as 1/3 and 2/3 × previous amount, producing a decrease of the sure amount in the next trials. **(C)** Behavioral results: binge drinkers (BD) had higher certainty equivalents compared with healthy volunteers (HV) corresponding to higher risk attitude in the BD subjects. Healthy volunteers in the reward condition made more risky choices in the highest risk (*p* = .1) compared with other levels of risk (*p* = .3, .5, .9) (*p* < .05) and in the loss condition made fewer risky choices in the higher risk (*p* = .1, .3) compared with other levels of risk (*p* = .5, .9) (*p* < .05). ⁎⁎*p* ≤ .05. M, magnitude; P, probability.

We used a novel adaptation of a risk-taking task without feedback, which included two independent counterbalanced sessions with reward and loss conditions (Figure 1A; Supplement 1). Participants chose between a gamble and a sure amount of money. For each condition, the gamble option included four randomly presented probabilities (*p* = .1, .3, .5, .9) and four expected values for each probability (£10, £50, £100, £500). For each probability and expected value, we used a staircase procedure in which the magnitude of the sure option was adjusted dependent on the choice in the previous trials, converging on the certainty equivalent (or indifference point between the risky and sure choices) after six successive choices (Figure 1B).

### Imaging Task

**Figure 2:**
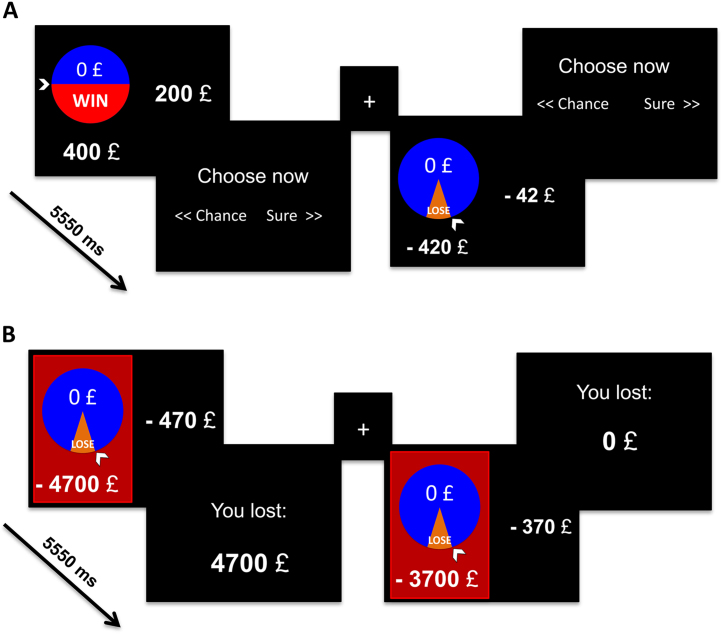
**(A)** The version of the task adapted for neuroimaging included counterbalanced reward and loss sessions with a high (.1) and a low (.5) risk probability and high expected values (£200–£500) for each probability. **(A)** Baseline task without feedback. Example of trials with low-risk reward trial (on the left) from reward session and high-loss loss trial from the loss session (on the right). **(B)** Task with feedback presentation. During the scanning, participants were shown the feedback of 10 trials in the high-loss condition (shown on red background) and then needed to make a choice in the same high-loss condition again (30 trials), when the feedback was not provided (identical to the baseline task shown in panel **A**).

The neuroimaging version of the task included choices between a sure amount or a gamble with low (.1) and moderate (.5) probabilities (Figure 2A; Supplement 1). The sure and gamble choices were of equal expected value (range £200–£500). The order of the task was counterbalanced for reward and loss conditions. Following the freely chosen baseline session, we specifically addressed the influence of feedback (FB) on high-risk (*p* = .1) loss choices.

Subjects were cued to choose the risky option in the FB trials (Figure 2B), during which 10% of trials (corresponding to *p* = .1) were associated with the loss of a large amount of money. Subjects then performed a post-FB session in which they chose between sure and gamble choices similar to the baseline session without associated FB. In both the behavioral and imaging tasks, the subjects were told at the beginning of the task that they should play as though the outcomes were real. The computer would select one of the trials at the end of the task. If they chose the risky choice on this trial, the computer would compute the gamble and they would win/lose a proportion of the possible outcomes or win/lose nothing. During the FB trial, they were told that they would lose a proportion of the feedback or nothing. Subjects were paid £7.50 per hour and an additional £5 depending on performance.

### Statistical Analysis of Behavioral Data

All statistical analysis was performed using Statistical Package for Social Science (SPSS; IBM, Armonk, New York) version 21. Before analysis, all variables were tested for Gaussian distribution (Shapiro-Wilk test, *p* < .05) and were transformed with square root transformation if necessary. Outliers (more than 3 SD above group mean) were removed.

The behavioral data were analyzed using a mixed measures analysis of variance (ANOVA) with group as a between-subjects factor (healthy volunteers [HV] and BD), and valence (reward, loss), probability (.1, .3, .5, .9), and expected value as within-subjects factors. A mixed-measures ANOVA with group as a between-subjects factor (HV and BD) and within-subjects factors of risk (high and low) and valence (reward and loss) was used for analysis of the neuroimaging version of the task. For significant main or interaction effects, post hoc analysis was performed using Tukey’s test. Demographic data were analyzed using a two-sample *t* test. All statistical comparisons were considered significant at *p* ≤ .05.

### Neuroimaging

**Data Acquisition.** T2*-weighted echo-planar images were acquired with blood oxygen level-dependent (BOLD) contrast on a 3.0 Tesla magnetic resonance scanner (Trio; Siemens, Malvern, Pennsylvania) with a 32-channel head coil using a tilted plane acquisition. Thirty-nine interleaved slices were acquired (repetition time = 2.32 sec, echo time = 33 msec, 3 mm slice thickness, no gap).

T1-weighted structural images were co-registered with the mean echo-planar images and normalized to a standard T1 template. Echo planar image data were analyzed within a general linear model, using SPM8 (Wellcome Trust Centre for NeuroImaging, London, United Kingdom). The first five volumes of each session were discarded to allow for T1 equilibration effects. Preprocessing consisted of slice-timing correction, spatial realignment, normalization using the same transformation as structural images, and spatial smoothing using a Gaussian kernel with a full-width at half maximum of 8 mm. To correct for motion artifacts, subject-specific realignment parameters were included in the general linear model.

**Neural Activations.** The decision and choice phases were modeled as a boxcar function convolved with the hemodynamic response function based on the time of onset with durations of 4500 milliseconds and 1000 milliseconds, respectively. There was a jittered intertrial fixation point (see Supplement 1) in which subjects fixated on a central + sign with a jittered duration (275 to 1225 msec). Six linear contrasts of regression coefficients (baseline session: high-risk [*p* = .1] loss [HL], low-risk [*p* = .5] loss [LL], high-risk reward, low-risk reward; post-FB session: high-risk loss [FB] and choice) were computed at the subject level and analyzed at the group level using a random-effects analysis.

We used a 2 × 3 ANOVA (flexible factorial design) with the within-subject factors of risk (high and low) and valence (reward and loss) and the between-subjects factor of group. To assess the effect of feedback, a 2 × 2 ANOVA (flexible factorial design) with the within-subject factors of feedback and between-subjects factor of group was used. Activations above familywise error whole-brain corrected *p* < .05 were considered significant.

### Region of Interest Analysis

Based on a recent meta-analysis of neuronal processing of risk anticipation (29), we included the regions of interest (ROI) as follows: superior parietal cortex (SPC), dorsolateral prefrontal cortex (DLPFC), dorsomedial prefrontal cortex, anterior insular cortices, and lateral orbitofrontal cortex (LOFC) (29). The Montreal Neurological Institute coordinates of the ROI are provided in Table S1 in Supplement 1. Regions of interest were defined as 8-mm spheres. The regression coefficients (betas) were extracted from ROIs on an individual level from the contrast of interest using MarsBaR 0.43v toolbox and compared between groups using one-way ANOVA with Bonferroni correction for multiple comparisons. Correlation analyses were performed among relevant behavioral variables and regression coefficients using Pearson’s correlation coefficient *r*.

## Results

### Participants

Seventy healthy volunteers and 40 BD were enrolled in the behavioral study. Twenty-one HV and 21 BD participated in the neuroimaging part of the study on a separate session. The data on two BD were not included in the final neuroimaging analysis due to technical problems during scanning (failure of the button response box).

In both parts of the study, the groups were matched for age, gender, and IQ assessed by the National Adult Reading Test (30). Alcohol consumption in both groups was evaluated using the Alcohol Use Disorders Identification Test scale (31). The demographic data of subjects are listed in Table 1.

**Table 1:** Subjects’ Demographic Data

Behavioral Part of the Study	Healthy Volunteers (*n* = 70)	Binge Drinkers (*n* = 40)	*t*	*p*
Age	23.68 ± 3.84	23.66 ± 6.30	.018	.985
Male Subjects (%)^a^	57%	54%		.283^a^
Intellectual Quotient	118.91 ± 6.73	118.82 ± 4.38	−.638	.532
AUDIT	3.71 ± 2.66	16.18 ± 5.97	−11.229	.0001
Years of Alcohol Use	5.03 ± 4.30	6.89 ± 3.18	−1.987	.06
Age at Onset of Alcohol Drinking, Years	15.84 ± 2.12	15.39 ± 2.63	.713	.479
				
Neuroimaging Part of the Study	Healthy Volunteers (*n* = 21)	Binge Drinkers (*n* = 19)	*t*	*p*

Age	24.14 ± 3.13	23.21 ± 3.52	−.886	.381
Male Subjects (%)^a^	45%	40%		.474^a^
Intellectual Quotient	117.27 ± 4.42	116.66 ± 4.17	−.686	.581
AUDIT	4.260 ± 3.06	13.72 ± 4.68	5.549	.0001
Years of Alcohol Use	6.15 ± 5.81	8.69 ± 5.11	1.228	.208
Age at Onset of Alcohol Drinking, Years	15.10 ± 2.46	14.31 ± 3.46	−.625	.538

Data reported in mean ± SD.

AUDIT, Alcohol Use Disorders Identification Test.

a Chi-square test.

### Behavioral Performance in Risk-Choice Task

Certainty equivalents were compared using mixed-measures ANOVA. The certainty equivalence was determined using a staircase procedure as outlined in Figure 1B in which the sure amount varied depending on the subject’s previous two choices.

There was a main group effect (*F*_1,88_ = 4.57, *p* = .04) in which BD had higher certainty equivalents compared with HV, which correspond to higher risk attitude in the BD subjects (Figure 1C). There was a main effect of valence (*F*_1,88_ = 252.45, *p* < .0001) but not of expected value (*F*_3,86_ = 2.52, *p* = .06) or probability (*F*_3,86_ = 1.53, *p* = .21).

There was a group × probability interaction (*F*_3,86_ = 3.79, *p* = .01): post hoc analysis showed that the difference in probability was significant for HV (*p* = .02) but not for BD (*p* = .97). There was no group × valence interaction (*F*_3,86_ = 2.16, *p* = .15).

There was an interaction effect of group × probability × valence (*F*_9,80_ = 3.16, *p* = .03), which on post hoc analysis, was driven by significant differences in probability in HV but not BD in the loss conditions (*p* < .0001 and *p* = .83, respectively), with no differences in the reward condition (*p* = .13 and *p* = .41, respectively). On post hoc analysis, HV in the reward condition had higher risk attitude in the highest risk (*p* = .1) compared with other levels of risk (*p* = .3, .5, .9) (*p* < .05) and in the loss condition had lower risk attitude in the higher risk (*p* = .1, .3) compared with other levels of risk (*p* = .5, .9) (*p* < .05). In contrast, in BD, there were no differences in risky choices between levels of risk in both reward and loss conditions.

### Behavioral Performance in Neuroimaging Task

There was a main effect of group (*F*_1,38_ = 10.460, *p* = .003) in which BD made more risky choices that HV. There was a main effect of valence (*F*_1,38_ = 3.241, *p* = .048) but no main effect of risk (*F*_1,38_ = 1.363, *p* = .249). There was a group × valence interaction (*F*_1,38_ = 5.868, *p* = .020) and a valence × risk × group interaction (*F*_1,38_ = 9.067, *p* = .005).

We specifically examined the loss condition comparing BD and HV: BD performed more risky choices in the HL (% of risky choices, mean ± SD, BD: 58.71 ± 9.66; HV: 39.52 ± 8.16, *p* = .024) with no difference in other conditions—LL (mean ± SD, BD: 55.40 ± 5.78, HV: 58.09 ± 6.96, *p* = .77), high-risk reward (mean ± SD, BD: 53.71 ± 7.06, HV: 49.16 ± 5.69, *p* = .61), low-risk reward (mean ± SD, BD: 47.89 ± 6.65, HV: 32.14 ± 5.63, *p* = .081). There was no difference in response time (RT) between groups in any condition (all *p* > .05).

### Neuroimaging Results

**Figure 3:**
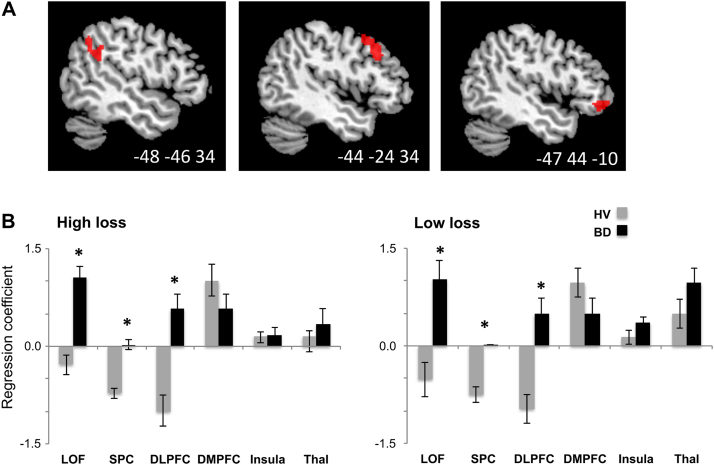
**(A)** Main functional magnetic resonance imaging clusters from group × valence interaction contrast (*p* < .05, familywise error correction for multiple comparisons): in order—superior parietal cortex, dorsolateral prefrontal cortex, and lateral orbitofrontal cortex. **(B**) Region of interest analysis: lateral orbitofrontal cortex (LOF); superior parietal cortex (SPC); dorsolateral prefrontal cortex (DLPFC); dorsomedial prefrontal cortex (DMPFC); anterior insular cortex (Insula); and thalamus (Thal). Reported in mean ± SD. ^⁎^*p* < .05. BD, binge drinkers; HV, healthy volunteers.

There was a main effect of group in which BD had greater activity in bilateral LOFC, SPC, and right medial prefrontal cortices and right superior frontal gyrus (Table S2 in Supplement 1). There was a group × valence interaction in the left LOFC, SPC, and DLPFC cortices (Figure 3A).

Region of interest analysis showed (Figure 3B) greater activity in HL and LL conditions in SPC (HL: *p* = .024; LL: *p* = .026), LOF (HL: *p* = .002; LL: *p* = .008), and DLPFC (HL: *p* = .030; LL: *p* = .012) in BD, with no difference in these regions in the reward conditions. There were no differences between the groups in BOLD activity in dorsomedial prefrontal cortex and insula for any task condition (all *p* > .05). There was no correlation between BOLD activity in these regions and amount of alcohol consumed in the groups (all *p* > .05).

### Behavioral Effects of Feedback Presentation

The FB arm of the task was specific to the HL condition and was assessed as the difference in the risky choices before and after FB presentation. Analysis of variance showed main effect of group (*F*_1,36_ = 9.82, *p* = .003), main effect of FB (*F*_1,36_ = 9.82, *p* = .003), and FB × group interaction (*F*_1,36_ = 6.22, *p* = .016).

**Figure 4:**
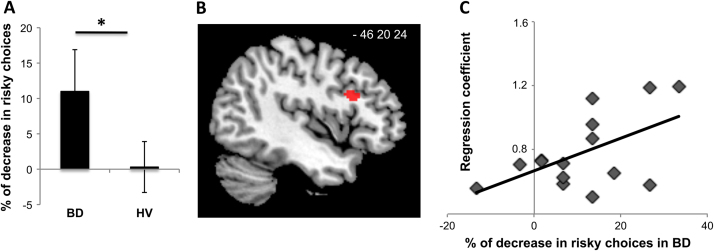
Behavioral and neuronal effect of feedback presentation. **(A)** Percentage of decrease in risky choices after feedback presentation; ⁎*p* = .03. **(B)** Main effect of feedback contrast with main cluster in left inferior frontal gyrus (familywise error correction for multiple comparisons). **(C)** Correlation of blood oxygen level-dependent signal from left inferior frontal gyrus within percentage of decrease in risky choices in binge drinkers (BD) after feedback presentation. Reported in mean ± SD. HV, healthy volunteers.

On post hoc analysis, BD showed a significant (*p* = .03) decrease in risky choices after FB presentation (mean % of decrease ± SD: 11.00 ± 5.90), which was not observed in HV (mean % of decrease ± SD: .31 ± 4.64) (Figure 4A). There was no difference in risky choices between the groups in HL after FB presentation (mean % of risky choices ± SD, BD: 45.40 ± 6.18; HV: 44.17 ± 5.19; *F*_1,36_ = .106, *p* = .747).

There was an effect of FB presentation on RT (*F*_1,36_ = 3.82, *p* = .049) without main effect of group (*F*_1,36_ = .43, *p* = .83). Post hoc analysis showed no difference between the groups in RT of risky choices (before FB: *F*_1,36_ = .11, *p* = .911; after FB: *F*_1,36_ = .154, *p* = .697) or sure choices (before FB: *F*_1,36_ = .397, *p* = .533; after FB: *F*_1,36_ = .498, *p* = .485) before or after FB presentation.

Using a within-group paired *t* test comparison, BD had significantly faster responses to sure choices after FB presentation (msec, mean ± SD, HL baseline: 467.99 ± 30.18; after FB: 421.00 ± 22.80, *t*_17_ = 2.88, *p* = .01) with no difference in RT in risky choices (msec, mean ± SD, HL baseline: 429.20 ± 30.18; after FB: 408.91 ± 20.32, *t*_15_ = 1.38, *p* = .186). There was no effect of FB presentation on RT in HV (risky choices: msec, mean ± SD, HL baseline: 432.50 ± 18.25; after FB: 412.77 ± 29.09, *t*_19_ = .57, *p* = .572; sure choices: msec, mean ± SD, HL baseline: 449.02 ± 18.42; after FB: 424.64 ± 20.77, *t*_19_ = 2.07, *p* = .057).

### Neuronal Effect of Feedback Presentation

The neuroimaging analysis showed a main effect of FB in the left inferior frontal gyrus (IFG) and inferior parietal cortex bilaterally (Table 2). The main effect of group also included DLPFC on the left and IFG and LOFC on the right (Table S2 in Supplement 1).

**Table 2:** Whole-Brain Main MRI Clusters

Region	Side	x	y	z	*Z* Score	Number Voxels	Corrected *p* Value
Group × Valence Interaction							
Lateral orbitofrontal cortex	L	−40	46	−6	4.94	47	.004
Superior parietal cortex	L	−52	−48	28	4.92	27	.009
Prefrontal dorsolateral cortex	L	−42	24	34	5.42	52	.004
Main Effect of Feedback							
Inferior frontal gyrus	L	−44	18	22	5.19	133	.002
Inferior parietal cortex	L	−50	−48	12	5.96	213	>.0001
	R	52	−38	12	4.83	93	.010

x, y, and z indicated in Montreal Neurological Institute space; *p* value from the familywise error correction for multiple comparisons.

L, left; MRI, magnetic resonance imaging; R, right.

Compared with HV, BD had a higher activity in left IFG (*F*_1,38_ = 4.706, *p* = .036), which was also positively correlated with percentage of decrease in risky choices in BD (*r* = .540, *p* = .038) (Figure 4B,C). There was no correlation of activity in left IFG with percentage of decrease in risky choices in HV (*r* = .002, *p* = .994). There were no differences between groups in activity of inferior parietal cortex (*F*_1,36_ = 1.063, *p* = .307).

## Discussion

We show that BD were more risk seeking when anticipating large unlikely losses compared with HV, an effect consistent with previous reports of decreased sensitivity to aversive reinforcement in BD (26). Furthermore, the neuroimaging results of an adapted version of the risk task demonstrated greater activity in cortical regions implicated in the processing of risk, including DLPFC, SPC, and LOFC in BD compared with HV. This is also in accordance with previous results in a study on incentive-related behaviors in BD compared with nondrinkers with hyperactivity in cortical regions (32).

We further introduced an experiential feedback with explicit exposure to the probability and magnitude of loss and show that risk attitude in BD can be influenced by feedback presentation. Following feedback, risk taking in BD decreased in the high-loss condition to the same level as that of HV. Whereas the BD were slower in their choices of the sure option before feedback compared with healthy volunteers, their response duration hastened for the sure option following feedback. Finally, on the neuronal level, the effect of feedback presentation was mediated via modulation of activity of left IFG, in which the greater activity was positively correlated with percentage of change in risky choices.

### Neuronal Mechanisms of Risk Seeking in BD

In BD, risk seeking in the high-loss condition was associated with hyperactivity in DLPFC, SPC, and LOFC. Numerous studies point to an association between activity in DLPFC, SPC, and LOFC with risky decisions 6, 33, 34 and judgments about probability and values 35, 36, 37, 38. Activity in DLPFC is suggested to play a role in the simplification of strategies of decision making under risky conditions, such as making choices focused only on the overall probability of winning (7). Activity in the SPC has been suggested to be related to the preference for risky choices (39). The LOFC, which purportedly tracks value in decision-making tasks 40, 41, had an increase in activity in both loss and reward conditions in BD. Such a pattern in LOFC activity might reflect the tendency to track the positive outcome (of gain or no loss) despite the risk of loss, consistent with the known role of the LOFC in updating reward-related associations regardless of outcomes (42). These results suggest a distortion of risk representation in the anticipation of risky losses in BD.

In this study, the amount of alcohol consumed did not correlate with BOLD activity in cortical regions. Other studies in alcohol-dependent and abstinent subjects also demonstrate greater risk taking, although they do not specifically address BD or dissociate reward and loss domains 17, 43, 44. Studies also suggest that binge drinkers have enhanced risk-taking behaviors in other domains, such as high-risk driving (45) and risky sexual intercourse (46). Exposure to alcohol decreases sensitivity to aversive reinforcement in both rodent 47, 48 and human studies (23), suggesting the possibility of a generalized decrease in sensitivity to the expectation of negative outcomes, secondary to the sequelae of alcohol use.

At baseline, BD subjects were predominantly more risk taking in the high-loss (low probability, high magnitude) condition compared with the healthy volunteers. This may be related to an enhancement of the diminishing marginal sensitivity with increasing loss magnitude in BD subjects (i.e., that BD subjects may subjectively value the higher magnitude loss less than the healthy volunteers). Further studies are required to assess this possibility.

### Effect of Explicit Exposure to the Probability and Magnitude of Loss

Compared with the baseline session, BD showed a significant decrease in high-loss risky choices following feedback presentation such that performance after feedback was the same as healthy volunteers. Binge drinkers were also slower to choose the sure choice at baseline and were faster to choose the sure choice following feedback. Overall, these findings suggest that in BD, the capacity to learn from outcomes is intact. Nonetheless, our study does not address whether this learning is related to probability, magnitude of the loss outcome, or an interaction of both.

Following the cued feedback in BD, the anticipation of HL risk was associated with an increase in activity in the left IFG. The IFG has been shown to code the subjective perception of the riskiness of an option (5) and risk aversion (49). The IFG is part of the cognitive control network (50) and a key locus in inhibitory control (51) and task switching 52, 53, suggesting a possible role for engagement of prefrontal inhibitory processes.

### Study Limitations

The behavioral and neuroimaging versions of the risk-choice task differed by design. The tasks differed in the number of risk probabilities and in the method in which risk taking was calculated: certainty equivalence using a step-wise procedure in which the reward values were positive and loss values were negative in the behavioral version and percentage of risky choices in the neuroimaging version. The tasks also differed in design (beads in a jar vs. pie chart). Such changes have been made deliberately to avoid the retest effect on behavioral performance. These task differences could explain some of the subtle behavioral differences in performance observed in the behavioral and neuroimaging parts of the study.

### Conclusions

These findings have important public health implications. Binge drinking is very common and characterized by significant negative physical, emotional, and financial consequences (54) and limited preventive interventions (55). Our findings suggest that the persistence of these behaviors might be related to impaired anticipation of the negative outcomes associated with risky choices. Here, we show that the experience of explicit feedback of probability and loss in BD subjects can potentially modify risk-taking attitude. Early intervention in BD is critical, as chronic repetitive exposure to ethanol in rodent models and in humans impairs the ability to learn to modify behavior 56, 57.
