# Supplementary material for: Neuronal Correlates of Risk-Seeking Attitudes to Anticipated Losses in Binge Drinkers
Source: Biol Psychiatry. 2014 Nov 1;76(9):717–24. doi: 10.1016/j.biopsych.2013.11.028 (PMC4192134; doi:10.1016/j.biopsych.2013.11.028)
Supplement: Supplementary file 1 — Supplementary Material [file mmc1.pdf]

Neuronal Correlates of Risk-Seeking Attitudes to Anticipated Losses in Binge Drinkers

Supplementary Information

Behavioral Tasks

The behavioral and neuroimaging tasks were programmed on a PC using E-prime professional v2.0 (www.pstnet.com/eprime.cfm) software.

The behavioral task included two independent sessions with Reward and Loss sessions. Subjects chose between a Gamble or Sure choice. Each session included four randomly presented risk probabilities (0.1, 0.3, 0.5 and 0.9) and four expected values (£10, £50, £100, £500) with a total of 96 trials/session. In both 'Reward' and 'Loss' sessions, choices were matched in terms of equivalence in expected value between gamble and sure options. Subjects first chose between a randomly selected probability and expected value Gamble and a Sure choice. The certainty equivalence was determined using a staircase procedure as outlined in Figure 1B in which the Sure amount varied depending on the subject's previous 2 choices. After 6 choices at the same probability and expected value, the certainty equivalence for that probability and expected value was calculated. The next randomly chosen probability and expected value Gamble and Sure choice was then presented.

Before each experimental session, the participants were first asked to read the task's instructions as follows, which could be reformulated orally if necessary: "You will see the jar with different proportions of red and blue balls. The proportion of balls in the jar will vary from trial to trial. You will also see a sure amount of money. You can pick a jar or a sure amount. If you pick a RED ball out of the jar, you will WIN (LOSE – in 'Loss' session) the amount indicated above the jar. Or, you can pick a sure amount in which you will WIN (LOSE) the amount indicated". The subjects were told at the beginning of the task that they should play as though the outcomes were real. The computer would select one of the trials at

the end of the task. If they chose the risky choice on this trial, the computer would compute the gamble and they would win/lose a proportion of the possible outcomes or win/lose nothing. If they chose the sure choice on this trial, they would receive/lose a proportion of the sure choice. Subjects underwent a practice trial of 6 choices so that they understood the step-wise nature of the task. The behavioral version of task was self-paced with unlimited time for response in each trial. Following the subjects' choice, the next trial was presented.

The neuroimaging version of the task included only 2 levels of risk – high (0.1) and low (0.5) in separate reward and loss sessions, with 60 trials/session (30 trials per condition – high loss, low loss, high reward and low reward). The likelihood of monetary Gain or Loss was presented as a pie chart with different colors: yellow for the loss and red for the gain of the amount of money indicated below the pie chart. The blue color in both sessions represented the likelihood of having 'nothing'. The sure choice was indicated as amount of money to gain or lose.

In both the behavioral and imaging tasks, participants were told that they would make a series of decisions and that they would win or lose a proportion of either the sure choice or a computer generated gamble outcome based on a randomly chosen trial. At the end of experiments, all participants received the equal payment of £15 for their performance in the task.

For the neuroimaging experiments, the participants were trained on a practice version of the task outside the scanner before performing the test session within the scanner. The participants were told that they should press the key of their chosen option only when they saw the 'choose now!' instruction. For the feedback session, they were told that they should choose the risky option, but feedback was not provided during the testing session. During the scanning participants were shown the feedback of 10 trials in the high-loss condition with a

forced-choice of the risky option. This was followed by the same high-loss condition without feedback in which they made freely chosen options (30 trials) identical to the baseline task.

For each trial, the duration of the decision-making stage was 4500 ms, with a choice stage of 1000 ms. Random time intervals (jitters), drawn from a uniform distribution between 275 and 1225 ms, were inserted between trials to ensure better sampling of the hemodynamic response and to avoid fatigue that can arise from a monotonous pace. The mean duration of task was 15 min. The echo-planar imaging acquisition was stopped upon the task completion.

Table S1. Montreal Neurological Institute coordinates of the regions of interest

Region	Side	x	y	z
Superior parietal	L	- 40	-54	42
Dorsolateral prefrontal	L	- 38	22	36
Dorsomedial prefrontal	L	- 4	30	32
Anterior insular cortex	L	- 30	20	8
Lateral orbitofrontal cortex	L	- 42	- 44	10

L, left.

Table S2. Main magnetic resonance imaging cluster

Region	Side	x	y	z	Z score	No voxels	*p*-value (corrected)
**Main Effect of Group in Baseline Task**							
Superior temporal gyrus	L	- 64	-22	4	5.54	48	0.004
Superior frontal gyrus	L	- 44	24	34	4.59	18	0.030
Superior parietal cortex	L	- 40	- 56	34	5.69	269	< 0.0001
	R	42	- 50	18	5.30	29	0.002
Lateral orbitofrontal cortex	R	42	26	-12	5.13	38	0.006
	L	-40	22	-22	5.12	46	0.005
Medial prefrontal cortex	R	6	48	32	4.92	17	0.021
**Main Effect of Group in High Loss Task after Feedback Presentation**							
Dorsolateral prefrontal cortex	L	- 46	26	42	4.60	37	0.026
Inferior frontal gyrus	R	54	26	8	4.51	22	0.037
Lateral orbitofrontal cortex	R	30	28	-20	4.45	6	0.046

x, y and z indicated in Montreal Neurological Institute space; *p*-value from the family-wise error correction for multiple comparisons.
L, left; R, right.

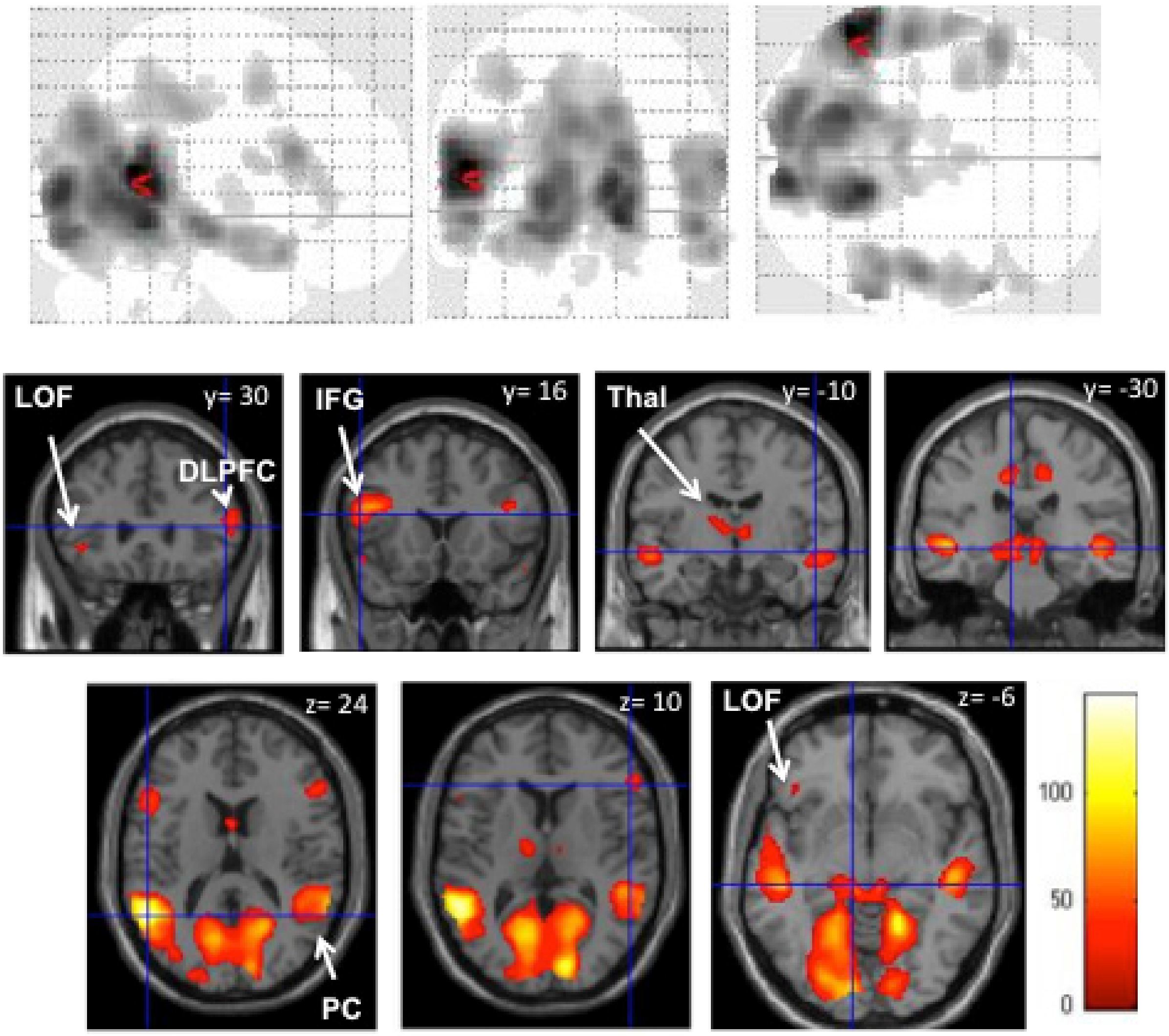

Figure S1. Main effect of task. The first row: The glass brain showing all main clusters (p = 0.05, family-wise error correction for multiple comparisons). The second and the third row: Main clusters of interest presented in coronal and horizontal views. DLPFC, dorsolateral prefrontal cortex; IFG, inferior frontal gyrus; LOF, lateral orbitofrontal cortex; PC, parietal cortex; Thal, thalamus.
